# Seroprevalence of hepatitis C virus in Jinan, China, 2008–2020

**DOI:** 10.1186/s40001-023-01063-0

**Published:** 2023-03-09

**Authors:** Mingjie Xu, Fang Liu, Qianqian Zhao, Yunying Zhou, Yuanmei Zhuang, Mingyu Ji

**Affiliations:** 1 Medical Research & Laboratory Diagnostic Center, Jinan Central Hospital Affiliated to Shandong First Medical University and Shandong Academy of Medical Sciences, 105 Jiefang Road, Jinan, 250013 Shandong People’s Republic of China; 2 Medical Research & Laboratory Diagnostic Center, Jinan Central Hospital Affiliated to Shandong University, Jinan, 250013 Shandong People’s Republic of China; 3 Shandong LaiBo Biotechnology Co., Ltd., Jinan, 250101 Shandong People’s Republic of China; 4 Department of Nephrology and Blood Purification Center, Jinan City Central Hospital Affiliated to Shandong University, Jinan, 250013 Shandong People’s Republic of China

**Keywords:** Hepatitis C virus, Seroprevalence, Prevalence

## Abstract

**Background:**

The updated estimates of hepatitis C virus (HCV) seroprevalence are critical for developing strategies to manage or eliminate HCV infection.

**Methods:**

A comprehensive study on HCV seroprevalence was conducted among 365,210 patients at Jinan Central Hospital, China, from 2008 to 2020. The patients were tested for anti-HCV, HCV core antigen, hepatitis B surface antigen, syphilis antibody, human immunodeficiency virus antigen + antibody, antihepatitis A virus IgM, and antihepatitis E virus IgM.

**Results:**

HCV seroprevalence was 0.79% and was related to age. HCV seropositivity was lower in children (aged  < 18 years) than in adults (aged  ≥ 18 years) (0.15% vs. 0.81%). High HCV prevalence was reported in adults aged  ≥ 41 years, and HCV seropositivity in those aged 41–80 years accounted for 74.56% of all seropositive individuals. Notably, the rate of HCV–HIV coinfection was 0. HCV seroprevalence was considerably higher in patients at the Kidney Disease Unit and Dialysis Department than in those at other departments (inpatient or outpatient).

**Conclusions:**

HCV seroprevalence was lower in Jinan region but higher in patients at the Kidney Disease Unit and Dialysis Department, especially in those undergoing hemodialysis.

**Supplementary Information:**

The online version contains supplementary material available at 10.1186/s40001-023-01063-0.

## Background

Hepatitis C virus (HCV) infection remains a serious health problem worldwide. Without appropriate antiviral treatment, 20–30% of patients with HCV infection will develop cirrhosis after 10–20 years, and 2–7% of these patients will develop primary liver cancer [1]. As direct-acting antiviral (DAA) therapy is widely used to control HCV prevalence, approximately 95% of patients with HCV infection could be cured. Consequently, the World Health Organization (WHO) has set a goal of eliminating viral hepatitis worldwide by 2030 [2]. The aim of this strategy is to reduce the numbers of new HCV infections and deaths by increasing the diagnostic rate to 90% and treating 80% of diagnosed patients. HCV infection can be eliminated in the next 10 years with focused strategies to screen and cure current infections as well as prevent new infections [2, 3]. However, strategies to eliminate HCV infection require a good understanding of the number of HCV infections.

Previous studies have reported global, regional, and national prevalence estimates of HCV infection. The total global HCV prevalence is estimated to be 2.5%, and the HCV prevalence in China is 1.3% [4]. According to the survey data of the China Center for Disease Control and Prevention, the prevalence of anti-HCV in the central region of China (0.67%) is slightly higher than that of the eastern and western regions (0.37% and 0.31%), and the prevalence of anti-HCV in the north (0.53%) is significantly higher than that of the southern region (0.29%). In this research, there is an increase from 2008 to 2013, decrease from 2014 to 2020, this may be related to the increased sensitivity of testing level, screening intensity and DAA treatment in Jinan. Updated estimate of anti-HCV prevalence is critical for developing strategies to manage or eliminate HCV infection. To date, no studies have analyzed HCV prevalence in Jinan (the capital city of Shandong province) at the regional level. Therefore, this study aimed to analyze the prevalence of HCV in Jinan, China, from 2008 to 2020.

## Methods

### Ethical statement

The study protocol was reviewed and approved by the Bioethics Committee of Jinan Central Hospital. Experiments were performed in accordance with the Bioethics Committee of Jinan Central Hospital’s guidelines and regulations. Written informed consent was obtained from all patients.

### Study population

From January 1, 2008 to December 31, 2020, 365,210 patients underwent anti-HCV IgG (anti-HCV) test and were admitted to Jinan Central Hospital in Jinan, Shangdong Province, China. Basic demographic data (age and sex) of the patients were collected.

### Laboratory assays

Sera samples were tested for anti-HCV using automated CMIA (Lai Bo Biotechnology, Inc. Jinan, China). The samples were considered positive when the results exceeded the cutoff (s/co) value of 1.00 (according to the manufacturer’s recommendations). The positive samples were subsequently tested for HCV core antigen (HCV cAg; Lai Bo Biotechnology, Inc. Jinan, China), hepatitis B surface antigen (HBsAg, SYSMEX CORPORATION, Janpan), HIV antigen + antibody (Ag + Ab; SYSMEX CORPORATION, Janpan), syphilis antibody (anti-TP; Lai Bo Biotechnology, Inc. Jinan, China), anti-hepatitis A virus (HAV) IgM, and anti-hepatitis E virus (HEV) IgM (Xiamen Innodx Biotech Co., Ltd., Xiamen, China).

### HCV prevalence in adults and children

Most studies have reported HCV prevalence in the adult population. In this study, the term “adult” referred to all individuals aged  ≥ 18 years. There are relatively few reports on HCV prevalence in children (aged  < 18 years). Herein, HCV prevalence in children (aged  < 18 years) from 2008 to 2020 was used to estimate the overall prevalence in children. When a study included the data regarding children, the prevalence in adults was calculated using the reported prevalence by age groups. In addition, adults were categorized into groups using 10-year intervals, and only individuals in the 18–30 year group had a 12-year interval.

### Statistical analysis

Statistical analyses were conducted using GraphPad Prism 8.0 statistics software. The Chi-square trend test (linear-by-linear association) was applied to calculate the *P* value for the seroprevalence of HCV in each year from 2008 to 2020. The Chi-square trend test (linear-by-linear association) to calculate the *P* value for the seroprevalence of HCV in each age group in each year from 2008 to 2020.The Chi-square test (Fisher’s Exact Test) to compare the HCV seroprevalence between children and adults. A *p* < 0.05 was considered statistically significant.

## Results

### HCV seroprevalence from 2008 to 2020

**Fig. 1:**
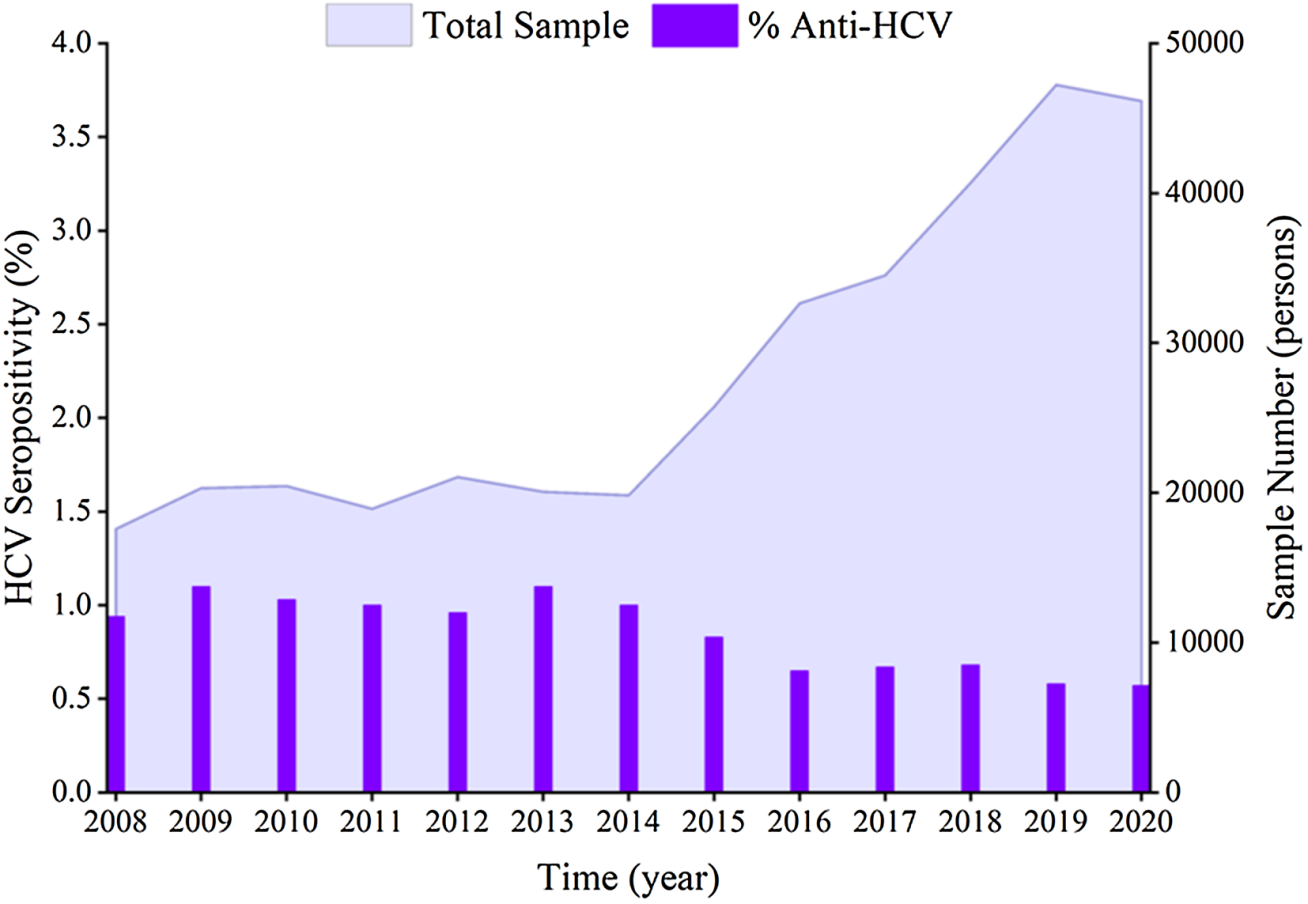
HCV seroprevalence in different years. The bar charts show HCV seroprevalence in different years in 2008–2020 at Jinan Central Hospital, Jinan, China. The area behind the bar chart represents the sample number in each year

From 2008 to 2020, the HCV seroprevalence was 0.79% in 365,210 patients (2885 positive vs. 362,325 negative samples). The HCV seroprevalences in the populations of 17,584, 20,310, 20,441, 18,925, 21,057, 20,066, 19,830, 25,750, 32,645, 34,515, 40,697, 47,235, and 46,155 individuals were 0.94%, 1.10%, 1.03%, 1.00%, 0.96%, 1.10%, 1.00%, 0.83%, 0.65%, 0.67%, 0.68%, 0.58%, and 0.57%, respectively. There is an increase from 2008 to 2013, decrease from 2014 to 2020(*χ*^2^ = 186.7, *P* < 0.0001) (Fig. 1).

### HCV seroprevalences in children and adults

**Fig. 2:**
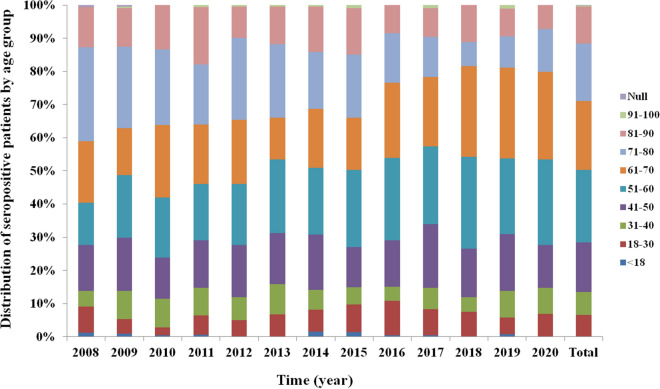
HCV seroprevalence in different age groups in 2008–2020. Data were categorized into age groups using 10-year intervals, except for those individuals aged 18–30 years, for which a 12-year interval was used. The bar graphs show the ration of HCV seropositivity. Null: patients did not provide individual age

The estimated HCV seroprevalences in children and adults from 2008 to 2020 are shown in Fig. 2. The HCV seroprevalence in children (aged  < 18 years) was estimated to be 0.15% (16/10854), which was lower than the overall HCV seroprevalence of 0.79%. The HCV seroprevalence among adults (aged  ≥ 18 years) was 0.81% (2,867/354,354), which was slightly higher than the overall HCV prevalence. The HCV seroprevalence in adults was highter than in children (*χ*^2^ = 58.87, *P* < 0.0001). In addition, studies reporting HCV seroprevalence in adults by age group were available. Adults were categorized into groups according to 10-year intervals, and only adults aged 18–30 years were categorized according to a 12-year interval. When compared with the adult group, the HCV seroprevalence was low in patients aged  < 40 (2.38–10.33%) and  > 90 (0.45–1.09%) years. The seroprevalence slightly increased in patients in the age groups of 41–50 (12.38–15.84%), 51–60 (12.65–22.17%), 61–70 (12.67–21.90%), and 81–90 (9.41–17.46%) years; however, it dramatically increased to 17.99%–28.31% in patients in the age group of 71–80 years from 2008 to 2013. When compared with the results obtained during 2008–2013, the seroprevalence was relatively low in young adults (≤ 40 years) and those aged  > 90 years and high in those aged  ≥ 40 years from 2014 to 2018, with annual peak prevalence of 20.20%, 23.26%, 24.88%, 23.48%, and 27.70% in the age group of 51–60 years, respectively. The peak prevalences in the age group of 61–70 years were 27.27% and 26.52% in 2019 and 2020, respectively. The *P* value for the seroprevalence of HCV in each age group in each year from 2008 to 2020 is showed in Table 1. Unfortunately, two patients did not provide their individual ages and accounted for 0.07% of the total positive samples (2,885).

**Table 1:** The P-value for the seroprevalence of HCV in each age group in each year from 2008 to 2020

Age	*χ*^2^-value	*P*-value	Statistically significant
< 18	12.10	0.4376	No
18–30	13.43	0.3386	No
31–40	40.57	< 0.0001	Yes
41–50	32.25	0.0013	Yes
51–60	38.29	0.0001	Yes
61–70	65.80	< 0.0001	Yes
71–80	137.00	< 0.0001	Yes
81–90	74.94	< 0.0001	Yes
91–100	Null	Null	Null

Null: Chi-square test could not be performed in 91–100 group, because the number of seropositives was 0 in 2008, 2010, 2014, 2016, 2018, 2020. Chi-square calculations are only valid when all expected values are greater than 1.0 and at least 20% of the expected values are greater than 5. These conditions have not been met, and thus the chi-square calculations are not valid

### Sex distribution of HCV seroprevalence from 2008 to 2020

**Fig. 3:**
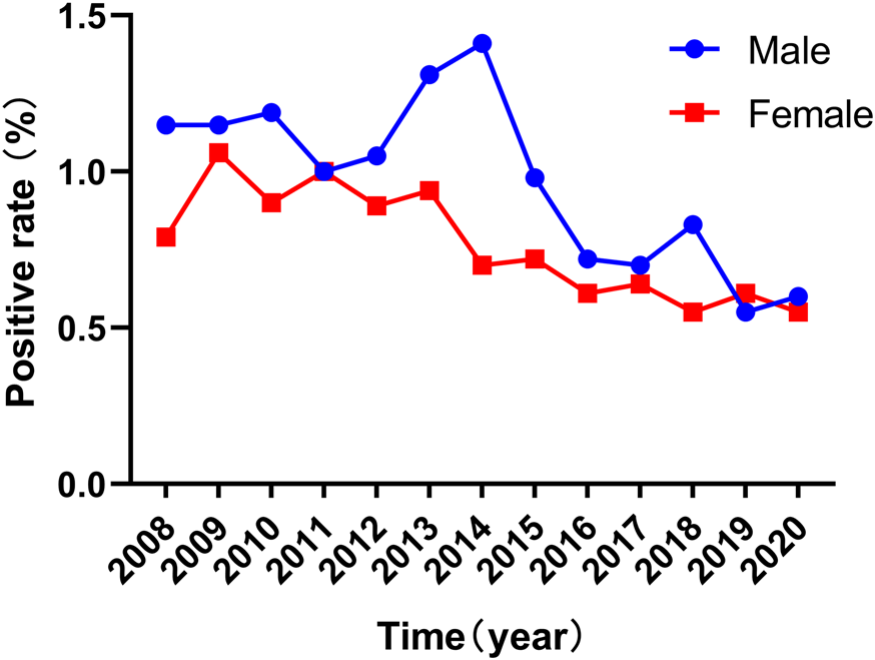
The gender distribution and HCV seroprevalence. 94/72, 122/102, 112/98, 86/103, 96/106, 116/105, 117/81, 113/102, 97/116, 111/119, 164/114, 130/145, 139/125 in 2008–2020 respectively. Data were showed in male/female

The sex distribution of HCV seroprevalence is shown in Fig. 3. There were differences in HCV seroprevalence between males and females in 2008, 2010, 2013, 2014, 2015, 2016, 2017, 2018 except in 2009, 2011, 2012, 2019, 2020 (data are showed in Table 2).

**Table 2:** The difference between male and female in HCV seroprevalence in each year from 2008 to 2020

Collected year	Male		Female		*χ*^2^-value	*P*-value	Statistically significant
	Positive	Negative	Positive	Negative			
2008	94	8065	72	9023	5.8650	0.0154	Yes
2009	122	10443	102	9561	0.4534	0.5007	No
2010	112	9294	98	10776	4.1250	0.0423	Yes
2011	86	8503	103	10157	0.0003	0.9857	No
2012	96	9034	106	11773	1.3730	0.2413	No
2013	116	8725	105	11085	6.3220	0.0119	Yes
2014	117	8191	81	11423	24.180	< 0.0001	Yes
2015	113	11362	102	14160	5.5780	0.0182	Yes
2016	97	13390	116	19021	1.5590	0.2118	Yes
2017	111	15849	119	18409	0.3666	0.5449	Yes
2018	164	19709	114	20690	11.510	0.0007	Yes
2019	130	23406	145	23546	0.7269	0.3939	No
2020	139	23183	125	22687	0.4679	0.4940	No

### HCV coinfection with other pathogens from 2008 to 2020

Overall, 2,771 patients with HCV seropositivity were subsequently tested for HCV cAg, HBsAg, HIV Ag + Ab, anti-TP IgG, anti-HAV IgM, and anti-HEV IgM. The positivity rates of HCV cAg, HBsAg, anti-TP IgG, anti-HAV IgM, and anti-HEV IgM were 12.38% (343/2,771), 4.26% (118/2,771), 0.79% (22/2,771), 0.40% (11/2,771), and 0.04% (1/2,771), respectively. Notably, the rate of HCV–HIV coinfection was 0. In total, 114 patients with HCV seropositivity voluntarily denied testing for other viral infection markers, accounting for 3.95% of the total positive samples (*n* = 2885). The data regarding the coexistence of HCV cAg, HBsAg, HIV Ag + Ab, anti-TP IgG, anti-HAV, and anti-HEV infection serological markers are shown in Table 3.

**Table 3:** Distribution of others viral infection markers prevalence in HCV seroprevalence in 2008–2020

Collected Year	HCV cAg ( +)	HBsAg ( +)	TP ( +)	HIV ( +)	HAV ( +)	HEV ( +)	NULL
2008	8	8	0	0	1	0	5
2009	15	8	0	0	0	0	3
2010	10	12	0	0	0	0	1
2011	9	5	1	0	0	0	5
2012	15	4	1	0	0	0	9
2013	37	4	0	0	3	0	7
2014	7	8	1	0	1	0	8
2015	16	7	3	0	0	1	7
2016	53	9	4	0	3	0	7
2017	59	9	2	0	0	0	15
2018	54	16	4	0	2	0	18
2019	30	15	3	0	1	0	19
2020	30	13	3	0	0	0	5
Total	343	118	22	0	11	1	114

Null: patients voluntarily gave up testing for other viral infection markers

### Outpatient and inpatient distribution of HCV seroprevalence from 2008 to 2020

**Fig. 4:**
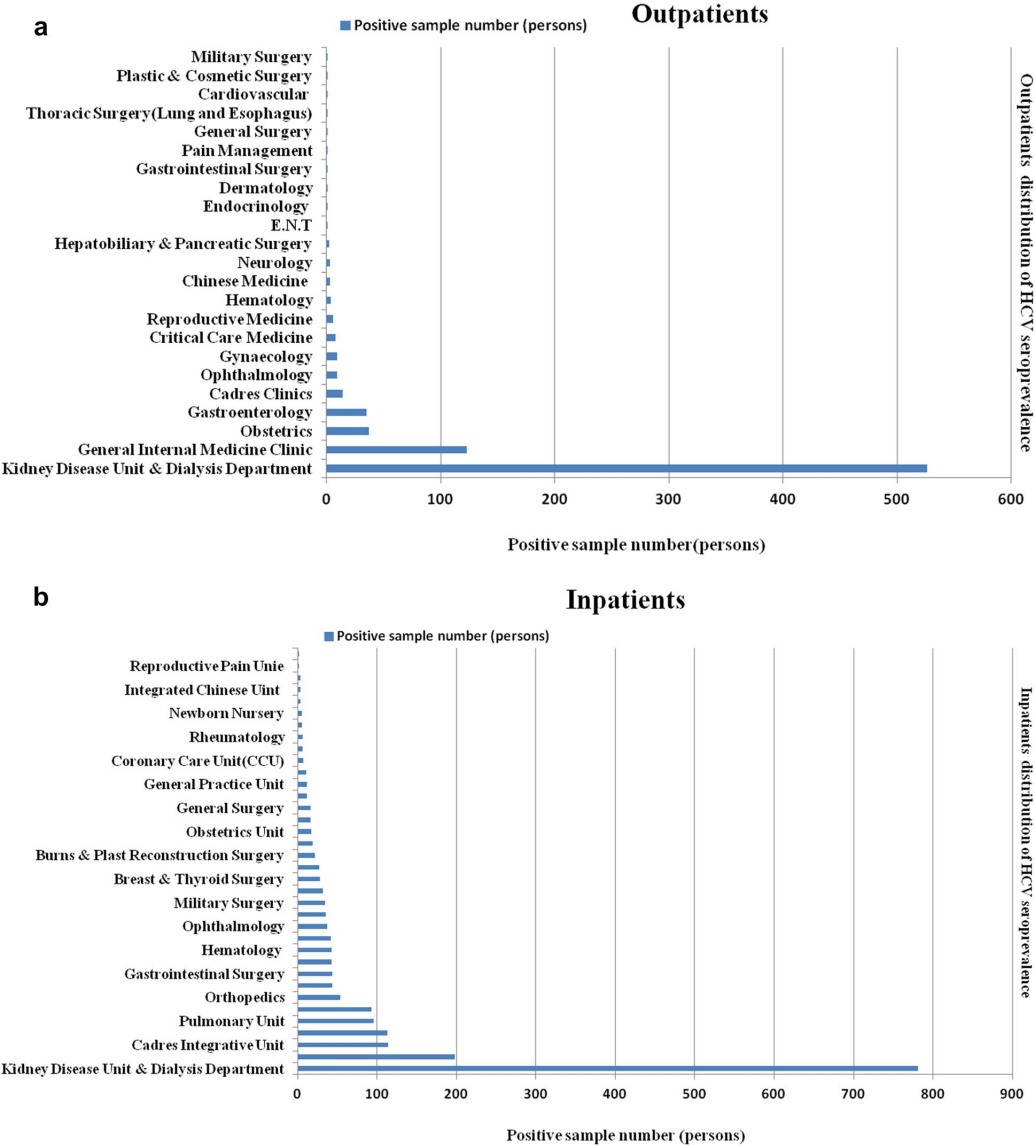
**A**. Outpatients distribution of HCV seroprevalence in 2008–2020.The departments that account for the total number of viral HCV positive infections in outpatients in the order of theirs contribution. **B**. Inpatients distribution of HCV seroprevalence in 2008–2020. The departments that account for the total number of viral HCV positive infections in inpatients in the order of theirs contribution

The overall HCV seroprevalence was 1.31% (789 positive vs. 59,274 negative samples) among 60,063 outpatients from 2008 to 2020. Patients at the Kidney Disease Unit and Dialysis Department accounted for 10.49% (526 vs. 5013) of all seropositive outpatients (Fig. 4a).

The HCV seroprevalence was 0.71% (2,032 positive vs. 284,216 negative samples) among 286,248 inpatients from 2008 to 2020. The top 10 departments that accounted for 77.76% (1,580 vs. 2,032) of the overall HCV seroprevalence in hospitalized patients were Kidney Disease Unit and Dialysis Department, Gastroenterology Unit, Cadres Integrative Unit, Neurology Unit, Pulmonary Unit, Cardiovascular Unit, Orthopedics, Hepatobiliary and Pancreatic Surgery, Gastrointestinal Surgery, and Urology Surgery. Patients at the Kidney Disease Unit and Dialysis Department accounted for 7.31% (781 vs. 10,682) of all seropositive inpatients (Fig. 4b).

The healthy screened and drug trial participants had HCV seroprevalences of 0.78% (36 positive vs. 4,597 negative samples) and 0.15% (21 positive vs. 14,045 negative samples), respectively. In addition, there were 200 participants who did not provide their departments at the time of medical treatment; among them, 7 were positive for HCV antibodies, accounting for 0.24% of the total positive samples (7 vs. 2,885). In addition, we have provided the amount patients of the different departments and the HCV positive numbers as a “supplementary table and summary statistics”.

## Discussion

HCV infections are globally distributed, and the total global HCV prevalence is estimated to be 2.5% (177.5 million adults with HCV infection) [4]. The HCV prevalence in China is 1.3% [4]. The updated estimates of HCV prevalence are critical for developing strategies to manage or eliminate HCV infection. The current retrospective analysis revealed the HCV seroprevalence of 0.79% in Jinan region, which was less than the total prevalence in China (1.3%).

There were relatively few studies that included children, and most studies have used a broad age group (e.g., 0–20 years) to describe the prevalence in younger populations. This study used a different HCV seroprevalence among children to adults ratio based on the age distribution to provide a more accurate estimate. As expected, the HCV seroprevalence among children (aged  < 18 years) was low at 0.15%, indicating that public health has improved since the introduction of HCV mandatory screening and universal prevention measures related to the transmission of blood-borne diseases, consistent with the previous findings [5].

Our study showed that HCV seroprevalence was closely related to age and increased significantly with aging among adults, with the highest seroprevalences observed in the age groups of 51–60, 61–70, and 71–80 years and low seroprevalence in those aged  < 40 and  > 90 years. This trend is consistent with the HCV prevalence in most countries [6, 7]. Blood is the main transmission route of HCV, which was first discovered by Chiron in the United States in 1989. However, until 1992, HCV screening before receiving blood transfusion, blood products, or hemodialysis or organ transplantation was widely used worldwide. People who had received blood transfusion and hemodialysis, used nonsterilized dental instruments, or underwent endoscopy or invasive procedures are high-risk groups of HCV infection, and individuals in these groups are currently aging [8]. The immune system of older individuals is relatively weak, and most of them suffer from other diseases and have low tolerance to therapeutic drugs. HCV infection in such individuals may cause serious harm. Therefore, in the future, the monitoring of HCV infections in older individuals aged  > 60 years should be strengthened (Additional file 1: Table S1).

Regarding sex, previous studies have shown that the positivity rates of serum anti-HCV were significantly higher in men than in women [7, 9]. In the current study, there were no significant differences in HCV seroprevalence between men and women from 2008 to 2020. Although there were more men than women with HCV infection (1,497 men vs. 1,388 women), this difference was not statistically significant. The precise reasons for this finding remain unclear, but may be related to different lifestyles, such as male homosexuality, sharing of syringes and needles for drug injections, and tattoos, thereby putting men at higher risk of HCV infection.

Other potential risk factors associated with HCV infection were analyzed by testing for HCV cAg, HBsAg, HIV Ag + Ab, anti-TP IgG, anti-HAV IgM, and anti-HEV IgM. Although HCV RNA testing is the gold standard for diagnosing HCV infection, it is expensive and requires a well-equipped professional laboratory. HCV RNA testing is challenging in regions with limited resources. The HCV core protein has approximately 190 amino acids, and its conserved amino acid sequence also plays a crucial role in virus proliferation and pathogenesis [10]. Therefore, HCV cAg detection could be a valuable alternative to HCV RNA detection. This study revealed that 343 individuals who were double positive for HCV-Ab and HCV cAg markers, accounting for 12.38% of the total positive samples, were considered to have active infections. This population is at risk of developing severe liver diseases; therefore, enhanced monitoring of infections in this cohort along with corresponding curative DAA treatment is a cost-effective approach and may reduce the future HCV burden.

HBV and HCV infection are strongly associated with liver failure, cirrhosis, and cancer [11]. According to the latest estimates, there are approximately 240 million individuals with chronic HBV infection and 110 million individuals with anti-HCV positivity worldwide [12]. The imbalance in disease burdens of hepatitis B and C is higher in low- and middle-income countries, particularly Asia and Africa [13, 14]. Screening, detection, and diagnosis of HBV and HCV infections are the key prerequisites for obtaining treatment and care services. Our study reported that 118 individuals were double positive for HCV-Ab and HBsAg markers, accounting for 4.26% of the total positive samples. Patients with HBV/HCV coinfection are significantly more likely to develop cirrhosis than those with HBV or HCV monoinfection [15, 16]. In this HCV/HBV coinfected population, routine HCV and HBV screening and early HBV vaccination should be improved to delay the occurrence and development of liver cirrhosis and reduce the disease burden. As reported in the previous study, early HBV vaccination of patients with HCV infection is important because the response may be better than that in the subsequent disease course, when cirrhosis develops [17].

China has experienced a dramatic resurgence of syphilis in recent years [18]. From 2011 to 2016, the total anti-TP positivity rate was 2.78% and HCV-TP coinfection rate was 0.06% [19]. Syphilis and hepatitis C have similar transmission routes, including blood transfusions, sexual transmission, and vertical transmission from the mother to child. The combined occurrence of local syphilis and hepatitis C in patients with syphilis is increasing every year, and our study found that 22 individuals were double positive for HCV-Ab and TP markers, accounting for 0.79% of the total positive samples. Combined with HCV infection, syphilis progresses more rapidly and has more serious consequences than simple syphilis. Therefore, the prevention, control, diagnosis, and treatment of syphilis combined with hepatitis C has become a clinical focus. Medical and health institutions should strengthen the detection of syphilis, hepatitis C, and other infectious diseases while providing infectious disease knowledge to the public to increase awareness of the disease and prevention strategies. HAV and HEV, which are primarily transmitted through the fecal–oral route, present as acute hepatitis and are responsible for most local epidemic outbreaks. The diagnosis is made by detecting serum HAV IgM, and anti-HEV IgM appears early during clinical illness. As reported, patients with chronic hepatitis C have a substantial risk of fulminant hepatitis and death if they subsequently contract HAV [20]. A previous study reported that HCV–HEV coinfection can potentially lead to a worse prognosis among Egyptians with chronic liver disease [9]. Among individuals with anti-HCV positivity, 0.40% and 0.04% of individuals were positive for anti-HAV IgM and anti-HEV IgM, respectively. Prevention of HEV infections, such as HAV, entails attention to general hygienic methods to avoid fecal–oral transmission of viruses. Furthermore, HAV and HEV vaccines have been developed and licensed in China. Strategies for screening viral hepatitis, vaccination, and post-vaccination testing need to be implemented in HCV–HAV and HCV–HEV coinfected patients. Because of shared transmission routes (drug injection and sexual transmission) between HCV and HIV infection [21, 22], the ratio of HCV is much higher in the HIV-infected population than in the general population [23, 24]. A surprising phenomenon was observed when we analyzed the correlation between HIV/HCV coinfected markers that no individuals were double positive for HCV-Ab and HIV markers. Therefore, we analyzed the factors that play roles in this phenomenon. Although 365,210 people participated in this study, it did not represent the general population of Jinan region. Moreover, some special groups involving sensitive personal privacy, such as high-risk sexual behavior, drug abuse, and homosexuality, were not fully included in the study and will eventually have some effect on the survey results. Moreover, there will be some biases. These considerations required us to focus on the population with the characteristics of the abovementioned high-risk factors in future research.

The current findings are consistent with previously reported findings showing that the prevalence of HCV infection is exceptionally high among dialysis and kidney transplant patients among outpatients and inpatients. The HCV seroprevalences were estimated to be 0.10.49% and 7.31% in outpatients and inpatients, respectively, at the Kidney Disease Unit and Dialysis Department. The worldwide prevalence of HCV antibody positivity in dialysis patients ranges from 2.7 to 68% depending on the country [25–30], and the prevalence of HCV infection substantially increases by as much as 90% in patients undergoing maintenance hemodialysis [31, 32].

In the past, inadequate screening of blood transfusion products and inadequate sterile medical techniques have led to the spread of HCV within the health care system, particularly in dialysis units. Currently, despite the introduction of strict hygienic precautions preventing infectious spread of HCV in dialysis settings, this infection remains prevalent among dialysis patients because of parenteral administration of drugs contaminated with traces of HCV-infected blood and the invisible contamination by blood from external surfaces and the hands of staff [33]. Thus, the application of basic hygiene precautions is crucial. These precautions include hand hygiene before contact with patients and after removal of gloves, changing gloves between patients or dialysis stations, preparing injectable drugs in a clean area, and cleaning and disinfecting surfaces of the hemodialysis environment before the next treatment session [34].

In a meta-analysis, Fabrizi et al. reported that the presence of anti-HCV antibody was an independent risk factor for death, with a relative risk of 1.57 in patients on maintenance dialysis. They also reported that dialysis patients with HCV infections were significantly more likely to develop hepatocellular carcinoma and liver cirrhosis [35]. HCV may also cause mixed cryoglobulinemic syndrome, which is a systemic vasculitis that can cause membranoproliferative glomerulonephritis [36].

HCV affects a large portion of the worldwide dialysis population and is associated with increased morbidity and mortality and lower quality of life. Accordingly, eradication of HCV infection in this specific population is highly recommended. All dialysis patients should be screened for HCV at the initiation of dialysis, and this should be repeated every 6 months. Once HCV antibody is detected, the HCV RNA viral load must be measured to confirm the presence of an active infection. Once the active infection is confirmed by a positive HCV RNA test result, the virus must be genotyped. Nephrologists and dialysis centers can prepare all patients for treatment according to their viral load and genotype. These steps will improve the chances of HCV treatment for patients receiving dialysis.

A major strength of this study was the large sample size of almost 365,210 patients throughout Shandong for 13 years. This large size is why we can presume that the HCV seroprevalence adequately represents the seroprevalence in the entire Shandong population. However, certain limitations of this large cohort study should be considered. The participants were primarily selected from one hospital, the patient population of which was used to represent the general population in Jinan. Selection bias was still possible, however. Second, plasma HCV RNA was not tested in patients who were anti-HCV-positive in this study because of limited funds, which made it difficult to distinguish active HCV infection. Therefore, further studies should be conducted in several hospitals in larger random samples with enhanced HCV screening in this patient cohort together with HCV RNA testing to accurately evaluate the prevalence of HCV in Jinan.

## Conclusion

This study showed a low HCV seroprevalence in Jinan region. However, the high HCV seroprevalence in patients at the Kidney Disease Unit and Dialysis Department, especially in those undergoing hemodialysis, indicates that this population is at risk of developing severe liver diseases. To achieve the WHO goal of eliminating HCV infection as a public health problem by 2030, medical staff and management personnel should primarily target HCV populations with high prevalence (e.g., hemodialysis group). We propose that HCV screening and DAA treatment in the diagnosed population should be increased to accelerate HCV elimination in the future.

## Supplementary Information

**Additional file1: Table S1. **The amount patients of the different departments and the HCV positive numbers.

## Data Availability

The data was collected from Jinan Central Hospital. The data that support the findings of this study are available from the corresponding author.

## Abbreviations

aa: Amino acid
Anti-HAV: Antibodies to hepatitis A virus
HBsAg: Hepatitis B surface antigen
anti-HCV: Antibodies to hepatitis C virus
anti-HEV: Antibodies to hepatitis E virus
HCV cAg: Core antigen of Hepatitis C virus
CMIA: Chemiluminescent microparticle immunoassay
HAV: Hepatitis A virus
HBV: Hepatitis B virus
HCV: Hepatitis C virus
HEV: Hepatitis E virus
HIV: Human immunodeficiency virus
IgG: Immunoglobulin G
IgM: Immunoglobulin M
RNA: Ribonucleic acid
s/co: Ratio of the sample optical density at 450 nm and the cutoff value
TP: *Treponema pallidum*
