# Supplementary material for: Seroprevalence of hepatitis C virus in Jinan, China, 2008–2020
Source: Eur J Med Res. 2023 Mar 9;28:112. doi: 10.1186/s40001-023-01063-0 (PMC9997000; doi:10.1186/s40001-023-01063-0)
Supplement: Supplementary file 1 — Additional file1: Table S1. The amount patients of the different departments and the HCV positive numbers. [file 40001_2023_1063_MOESM1_ESM.docx]

**Supplementary table The amount patients of the different departments and the HCV positive numbers.**

| **Departments** | **Positive** | **Negative** | **Positive rate(%)** |
| --- | --- | --- | --- |
| Kidney Disease Unit & Dialysis | 1307 | 14388 | 8.33* |
| General Practice Unit | 12 | 613 | 1.92 |
| Plastic & Cosmetic Surgery | 3 | 227 | 1.30 |
| Dental Clinic | 4 | 307 | 1.29 |
| Gastroenterology Unit | 233 | 20169 | 1.14 |
| Oncology Unit | 42 | 3785 | 1.10 |
| Hepatobiliary & Pancreatic Surgery | 46 | 4825 | 0.94 |
| Hematology | 47 | 5442 | 0.86 |
| General Surgery | 17 | 2087 | 0.81 |
| Cadres Integrative Unit | 128 | 16304 | 0.78 |
| Healthy screened | 36 | 4597 | 0.78 |
| Integrated Chinese Uint | 7 | 887 | 0.78 |
| Endocrinology | 13 | 1721 | 0.75 |
| Military Surgery | 36 | 5112 | 0.70 |
| Intensive Care Unit(ICU) | 35 | 5041 | 0.69 |
| Gastrointestinal Surgery | 45 | 6556 | 0.68 |
| Burns & Plast Reconstruction Surgery | 22 | 3219 | 0.68 |
| Rheumatology | 6 | 924 | 0.65 |
| Vascular Surgery | 32 | 4961 | 0.64 |
| Pulmonary Unit | 96 | 15039 | 0.63 |
| Thoracic Surgery | 20 | 3263 | 0.61 |
| Dermatology Unit | 6 | 994 | 0.60 |
| Neurology Unit | 116 | 19923 | 0.58 |
| Ophthalmology | 46 | 8045 | 0.57 |
| E、N、T Unit | 17 | 3025 | 0.56 |
| Anorectal Unit | 11 | 1951 | 0.56 |
| Urology Surgery | 43 | 7764 | 0.55 |
| Cardiac Surgery | 4 | 756 | 0.53 |
| Infectious Disease Ward | 2 | 377 | 0.53 |
| Cardiovascular Unit | 94 | 20349 | 0.46 |
| Breast & Thyroid Surgery | 28 | 6549 | 0.43 |
| Orthopedics | 54 | 13044 | 0.41 |
| Pediatric Unit | 6 | 1949 | 0.31 |
| Obstetrics Unit | 54 | 18097 | 0.30 |
| Newborn Nursery | 5 | 1648 | 0.30 |
| Coronary Care Unit(CCU) | 7 | 2421 | 0.29 |
| Reproductive Pain Unit | 3 | 1063 | 0.28 |
| Gynecology Unit | 45 | 30663 | 0.15 |
| Drug trials | 21 | 14045 | 0.15 |
| General Internal Medicine Clinic | 123 | 84819 | 0.14 |
| Reproductive Medicine | 6 | 5183 | 0.12 |
| Total# | 2878 | 365010 | 0.78 |

*P value <0.0001

#: There were 200 participants who did not provide the departments at the time of medical treatment, and among of them, 7 were positive for HCV antibodies.
